# Motivations and challenges experienced among locums working in Norwegian rural general practice: a reflexive thematic analysis

**DOI:** 10.1080/02813432.2026.2679616

**Published:** 2026-06-01

**Authors:** Henrik Hansen Wallumrød, Birgit Abelsen, Anette Fosse, Martin Bruusgaard Harbitz

**Affiliations:** ^a^ Department of Community Medicine, Faculty of Health Sciences, UiT The Arctic University of Norway, Tromsø; ^b^ Department of Community Medicine, Faculty of Health Sciences, University of Tromsø, Tromsø, Norway

**Keywords:** Rural, general practice, locum, primary healthcare, continuity of care

## Abstract

**Purpose:**

To further our understanding of temporary work and rural healthcare services by exploring doctor’s motivations for, and experiences with, locum work in Norwegian rural general practice.

**Methods:**

Individual semi-structured interviews were conducted with general practice locums who were currently working or had recently worked in a rural area. Audio recordings from the interviews were transcribed verbatim and analysed using reflexive thematic analysis.

**Results:**

Locums in rural general practice were often recruited by third parties (agencies or colleagues). Early career locums usually found it easier to use agencies. Most of the locums were attracted to temporary work for the work-related autonomy, work-life balance, interesting locations (locum tourism) and attractive salaries. Experienced locums emphasised work-life balance and clinically focused work, while early career locums often sought to accumulate work experience. All locums reported being under high expectations to perform independently, often with minimal formal support. Early career locums expressed a desire for formal support to compensate for their limited experience. To maintain continuity of care, locums used longer consultations and wrote detailed notes in the electronic patient records.

**Conclusion:**

In light of recruitment and retention-challenges in general practice it is worth reflecting on the results that many locums were attracted to rural general practice by the combination of high salaries, better work-life balance and time for clinically focused work. In addition, locum work offered valuable clinical experience for early career locums. On the other hand, locums experienced discontinuity of patient care, high expectations to perform, considerable responsibility and minimal formal support. There is a need to explore how to improve formal support, such as standardising introductory programmes and providing mentoring sessions, especially for early career locums, and how to adapt work routines to strengthen continuity. In light of our results, we question the wisdom of maintaining lower qualification requirements for doctors taking temporary contracts as a measure to increase locum recruitment in rural and remote areas.

## Introduction

The use of locums (doctors covering temporary vacancies) has been increasing steadily in order to provide care to patients on regular general practitioner (GP) patient lists in Norway [1]. Of all the hours worked by doctors in Norwegian general practice from 2008 until 2021, locums accounted for 15% [2].

The use of locums is important to ensure sufficient access to care for patients during short- or long-term vacancies. Additionally, locums might bring valuable skills, new perspectives and offer flexible adaptations to challenging staffing situations to mitigate the workload of regular staff [3,4]. However, the use of locums has also been associated with challenges, such as reduced continuity of care [4,5], increased likelihood of prescribing antibiotics [6–9] and more use by patients of out-of-hours care [8]. Yet there is limited evidence to suggest that locums represent a higher risk to patient safety than regular doctors [10–13].

General practices in rural and remote areas often experience high turnover of GPs. Frequent use of locums contributes to fragmentation of rural healthcare services by increasing the workload of regular staff, who are required to provide ad hoc supervision of inexperienced locums [14]. Locum doctors have a unique role in health care, and their perspective could provide valuable insights into rural general practice. However, to our knowledge, no studies have examined the perspectives of doctors performing locum work in rural general practice. Thus, we aimed to explore doctors’ motivations for, and experiences with locum work in Norwegian rural general practice through a qualitative approach.

## Materials and methods

### Study setting

#### The Norwegian general practitioner scheme

The Norwegian healthcare system is divided into specialist care provided by regional health trusts, and primary care organised by each municipality. GPs play a central role in primary care, providing 92% of all primary care consultations. They are often the patients’ first point of contact with the healthcare system and serve as gatekeepers to specialist healthcare services [15]. All residents in Norway have the right to be registered with a GP, i.e. on the list of that GP. The aim of this GP scheme is to provide all inhabitants of Norway with a regular GP and continuity of care [15]. Each municipality is mandated to ensure access to care for its inhabitants [15]. Therefore, lists that do not have a regular GP are often covered by locums to ensure access to care [15]. Approximately 90% of all lists without a designated regular GP have stable locum coverage, accounting for around 138,000 individuals, or about 2.5% of the Norwegian population.

Additionally, municipalities are obligated to ensure that the regular GPs they employ are either specialists in general practice or in the process of obtaining the qualification. However, for temporary GP vacancies of up to one year, specialist qualifications are not mandatory [16]. This results in a difference in qualification requirements between regular GPs and locums.

For comparison, Sweden allows doctors without specialisation (underläkare) to practice as locums [17]. However, it requires a time-limited permit, and the locum must have a supervisor and colleagues to support with local routines. Employers are also explicitly obligated to provide training for the locum [17]. Denmark and the United Kingdom, are examples of more restrictive models, where locums need to hold the same qualifications as regular GPs with exception of short term locum contracts [18,19].

#### Rural general practice

Statistics Norway [20] has classified all Norwegian municipalities based on their level of ‘centrality’. This classification is determined by travel time to services and workplaces, with an index ranging from ‘1’ (most central) to ‘6’ (least central). In this study, we define rural municipalities as those with a centrality level of 4, 5 or 6. Of Norway’s 5.6 million inhabitants, 29% reside in rural areas [21].

Rural healthcare is characterised by unique and interconnected challenges in terms of access to care, organisation of healthcare and the healthcare workforce [22]. In Norway, remote locations, adverse weather conditions, high staff workload, and patient reluctance to seek healthcare and locums have been identified as risk factor for patient safety in rural healthcare [14,23].

Additionally, globally, there is a demographic shift towards an increasingly elderly populations, with younger people leaving the rural areas for more urban areas [24]. In Norwegian rural practice this means more older patients, often with chronic conditions and multimorbidity [2,21]. As a consequence of long distances to specialist services, rural GPs provide more long-term complex care than urban GPs [25]. Furthermore, in general rural primary healthcare services experiences high GP turnover [22]. In Norway rural and remote areas have the highest proportion of long-term vacant GP lists [15]. Some Norwegian municipalities have utilised work-leave rotation schemes to increase recruitment and retainment of GPs. In a work-leave rotation, the GP alternates between intervals of work (often on site) and leave, where the GP often lives a long way from the surgery [26].

#### Continuity of care

Continuity is an essential aspect of general practice. Saultz [27] developed a hierarchical perspective, where continuity was divided into three levels: informational, longitudinal and interpersonal. The lowest level, informational continuity, involves a healthcare provider having access to relevant medical and social information about a patient. Longitudinal continuity is maintained through healthcare facilities that take responsibility for the care of a patient over time. Interpersonal continuity, the highest level, is when a patient has a particular healthcare provider whom the patient trusts and who has accumulated knowledge about the patient’s history. Disrupted continuity of care is associated with increased risk of patients using out-of-hours services, acute hospitalisations and increased mortality [28].

### Study design

Qualitative methods are appropriate for exploring lived experiences. We conducted 13 individual semi-structured interviews to explore the motivations and experiences of locums working in Norwegian rural general practice.

### Interview guide

The interview guide was developed based on the available literature on locum work in primary and secondary healthcare, and on the research team members’ experience and knowledge of general practice in rural Norway. There is little information available on locum work from the perspectives of locums, particularly in rural healthcare. Hence, we adopted an exploratory approach to the questions and included open-ended questions, investigating the participants’ motivations for taking locum work, the recruitment process, introduction and training, experiences of their working day, their social life during contracts and the terms and conditions of their locum contract (Online Appendix 1). The research team reviewed the interview guide continuously and added questions between interviews to explore relevant points of interest.

### Recruitment and participants

The locum participants were recruited through purposive and snowball sampling from rural municipalities. We contacted administrative healthcare leaders who contacted locums among their current or former (<12 months) staff. Eleven eligible participants responded, consented and participated. Additionally, two candidates were recruited through the Norwegian Centre for Rural Medicine network. We sought variation in contract duration, geographical location, locum experience, level of medical specialisation, locum recruitment methods, nationality, gender and age.

The participants were a mix between Norwegian and non-Norwegian locums (Table 1). The Norwegian locums consisted of a mix of specialists in general practice and non-specialists, experienced and inexperienced, younger and older, some had studied for their medical degree in Norway and some in EU/EEA countries. On the other hand, non-Norwegian locums were all relatively young (<39 years) and were not trained as specialists in general practice. Their medical training was either from their home countries or other EU/EEA countries. All non-Norwegians had either learned Norwegian to a conversational or fluid level or relied on an adapted version of their own Nordic language.

**Table 1.** Demographic data of the 13 locums doctors included in the study.

Demographic factor	Frequency
Gender	
Women	6
Men	7
Age	
<30	3
30–39	7
40–49	1
50–59	2
Nationality	
Norwegian	7
Nordic*	5
EU/EEA**	1
Specialisation in general practice	
Yes	3
No	7
Ongoing specialisation	3
Number of locum contracts	
1	5
2–5	4
6–9	1
>10	3

*Excluding Norwegians; **Excluding Nordics.

### Data generation and analysis

Data were collected between May and August 2024. The interviews were conducted *via* digital platforms and began with an introduction and gathering of demographic data. All interviews were held in Norwegian. Eight interviews were conducted by two interviewers, with the first author, HHW (MPH), acting as the primary interviewer while the co-authors, MBH (PhD, MD), AF (PhD, MD) and BA (Professor) alternated as the second interviewer. We collected a total of 663 min of interview audio, with a mean duration of 51 min for the 13 interviews. Five of the interviews were conducted by HHW alone. Audio recordings of the interviews were paired with field notes taken by HHW during and after interviews. The interviews were transcribed by a professional transcriber and the AI tool Klartekst (UiT The Artic University of Norway, 2024).

We analysed the data using reflexive thematic analysis [29]. This process consists of six steps: familiarisation with the dataset, coding, generating initial themes, reviewing and refining themes, defining and naming themes and writing up a report. The analysis was not guided by any specific theory, but we had the concept of continuity of care in mind throughout the research process.

Familiarisation was performed by all authors by reading through the transcripts. Codes were developed inductively by HHW and MBH: at first separately, followed by HHW and MBH comparing their codes, discussing the meaning and intention of each code and how they could be constructed as initial themes. These initial themes were formulated and presented to AF and BA. All authors discussed the initial themes over several meetings and provided feedback for each developing theme. For the fifth step, MBH reviewed all themes and ensured that they were anchored in the data. HHW translated the themes to English. All authors contributed to the final definition and naming of the themes and the subsequent drafting of the manuscript. The aim of involving several researchers was to increase the breadth and nuance of the data interpretation.

Reflexive thematic analysis embraces the subjectivity of the researcher(s) as an essential part of the analysis. MBH and AF are GP specialists and have worked for several years as rural GPs. BA has extensive experience in healthcare research with a particular focus on Norwegian general practice. MBH, AF and BA are trained in qualitative research methods. HHW is a PhD candidate researching the use of locums in Norwegian rural municipalities using quantitative and qualitative methods. The researchers share a concern about the increased use of locums and the potential loss of continuity in patient treatment. They therefore make efforts to increase knowledge about what motivates locums and how we can learn from their experiences to develop robust and sustainable rural healthcare services.

### Ethical considerations

All participants invited to this study received information about the background and purpose of the study, how the data would be handled, and their rights as participants. This enabled them to give informed consent to participate in the study. To preserve participants’ anonymity for publication demographic data were aggregated (Table 1). The plan for storage, handling and analysis was approved by the Norwegian Agency for Shared Services in Education and Research (Reference: 120360).

## Results

We generated four themes: (1) Entering and leaving rural locum work, (2) Good working conditions and how to get them, (3) High expectations and limited support and (4) Maintaining continuity in discontinuity. These themes were further divided into six additional sub-themes (Table 2). We noticed a difference between experienced locums and early career locums. However, we did not perform a comparative analysis but added this aspect as another dimension to the analysis.

**Table 2.** Overview of themes and sub-themes.

Themes	Sub-themes
Theme 1: Entering and leaving locum work	*Locum recruitment: fast agencies and old friends*
	*Barriers to long-term locum and regular GP work*
Theme 2: Good working conditions and how to get them	*Attractive working conditions: nature, freedom and high salaries*
	*How locums negotiated: picking and choosing between municipalities*
Theme 3: High expectations and limited support	*High expectations: poor communication and need for support*
	*Introduction to rural life: left alone with the keys, an emergency radio and off you go*
Theme 4: Maintaining continuity in discontinuity	*Barriers to continuity*
	*Strategies for maintaining continuity*

### Theme 1: entering and leaving rural locum work

#### Locum recruitment: fast agencies and old friends

The locums talked about different channels of recruitment such as locum agencies and their own social network. They said that locum agencies contacted them or offered digital sign-up platforms. The agencies studied the locums’ preferences for time, place and duration, and offered contracts shortly afterwards. Several locums mentioned that locum agencies supported them by arranging courses, financing their travelling and accommodation, and provided advice when locums applied for Norwegian medical authorisation. This participant explained how the agencies simplified the recruitment process for locums:

“*…The reason I chose an agency was because it was easy… Easy and simple. You get a kind of package deal, and so you take it.*” – Locum 2

However, more experienced locums were concerned that the locum agencies might not check for relevant clinical experience and sufficient knowledge of the Norwegian welfare system.

Some locums were recruited through their own social networks (by former locums). They were given advice about whom to contact and which municipality to prioritise. Experienced locums often had established networks informing them about locum contracts, but several had used agencies early in their locum career. Early career locums often worked with locum agencies as it was easy to obtain a contract, and the agencies offered higher salaries than municipal contracts.

### Barriers to long-term locum and regular GP work

Several young locums from abroad were frustrated at the difficulty of obtaining a long-term locum contract or permanent positions in Norway without specialisation in general practice. These locums felt obliged to leave and return to their home countries, where they planned to start their specialisations.

“*They wanted me to keep working at the surgery, but according to the law as it stands today I have to leave Norway as I have stayed here for more than a year* – Locum 2.

Several locums applied for positions as rural regular GPs in a work-leave rotation scheme, mostly in municipalities where they had worked and thrived as a locum. However, some participants explained that some locum agencies used clauses in contracts that prevented locums from taking a job directly as a locum or regular GP from any municipality where they had recently worked as a locum.

### Theme 2: good working conditions and how to get them

#### Attractive working conditions: nature, freedom and high salaries

The locums stated that they sought beautiful Norwegian nature, a good work–life balance and a high salary. Some locums estimated that they earned four times as much as a locum in Norway as they would as a resident doctor in their home country. Rural areas offered early career locums high salaries and relevant clinical experience for their future career. On the other hand, priorities for experienced locums were less stress and more time with patients than in their previous work as a regular GP, even though they often had to spend some time to rectify the negative consequences of discontinuity, as explained by this experienced locum:

“*There’s a lot of tidying up loose ends here… they haven’t done any blood tests for the past two years… people aren’t getting the care they should be getting. And I really enjoy doing that kind of work. And then I’ve got time to do it. Because in the countryside, things move at a slower pace, and that suits me fine, so I can really learn a lot and do a proper job with every single patient.*” – Locum 8

The locums stated that the location itself and its proximity to urban areas influenced the attractiveness of the contract. Some locums described having a ‘bucket list’ of locations they wanted to work in and experience. Additionally, one early career locum added that it was easier to obtain contracts in rural areas because of high competition for urban locum contracts.

The experienced locums said that regular work in healthcare led to stress and a guilty conscience when they tried to maintain a demanding balance between work and private life. One locum told about their experience in regular employment in the healthcare sector:

“*I often feel it’s been very rigid. And the feeling that you’re not allowed to have control over your own free time because you are ordered into duty. Or rather, pressured into duty, or pressured to overtime. I think that those things have been really unfair.* “– Locum 4

Locum work in rural areas allowed the locums to achieve a better work-life balance, either by having more time off when returning home after finishing the contract, or during a contract because of the working hours they had negotiated as expressed by the following locum:

*“I actually get to work as a GP in a way that is sustainable and meaningful. It feels like what I am doing is important, and it’s not killing me!”* – Locum 9

#### How locums negotiated: picking and choosing between municipalities

The locums spoke of how they negotiated with municipalities on their working conditions. Locums that negotiated conditions for themselves reported being able to influence their salary and overall working conditions, and felt they had the freedom to pick and choose between municipalities. Some wanted to work as much as possible, taking contracts where they were on-call during the entire contract (i.e. for some weeks), but most locums sought a work-life balance. Therefore, some only took contracts that offered regular surgery hours and refused out-of-hour duties during negotiations:

“*Autonomy has been very important. And it’s been very important not to be on call for emergencies, so I can just go to work and go home like everybody else, and not be ready to drop till the next day.*” – Locum 4

For some locums, the agency negotiated with the municipality on their behalf based on their preferences. In these cases, the locums often did not know the total amount of salary passed between the agency and the municipality. They received drafts of final contracts to review and approve. Some locums estimated that the agencies pocketed about 1/3 of the total amount. Furthermore, the contracts from locum agencies differed from contracts where the municipality hired the locums directly; the agency contracts often offered higher salaries. Several locums had experienced that another locum hired on a municipal contract would find this difference unfair and caused a conflict when trying to negotiate a salary matching what the agencies offered.

### Theme 3: high expectations and limited support

#### High expectations: poor communication and need for support

Several locums talked about high expectations from the municipality, which were sometimes unrealistic in relation to their clinical skills and working capacity. A few early career locums ended their contracts after only a short period, feeling that the overwhelming workload and lack of support was not conducive to safe patient care. Several experienced locums had handled complex patient cases or worked full night shifts in out-of-hours services without support. These locums stated that they were expected to function as an experienced GP specialist – which they were not.

*“It’s no secret. I did find that contract very hard, that’s why we ended up resigning rather than completing the contract. I felt that the pressure and the workload was really, really heavy. And I did not feel equipped for that.” –* Locum 13

They underlined that these expectations had not been clearly communicated to them when they were hired. Therefore, early career locums expressed a need for daily guidance and sought other doctors for help. However, if none were available, they turned to the general practice staff (medical secretaries and nurses) for advice, whom some referred to as ‘the gems of the surgery’. Several locums wanted more formal mentoring, though this was seldom offered. On the other hand, locums with specialisation in general practice did not mention any need for mentoring. A small number of locums mentioned benefitting from formal mentoring agreements:

“*And then I had a mentoring session once a week. And it was very constructive. But now I’ve heard the municipality no longer has those sessions for new temporary staff*.” – Locum 3

#### Introduction to rural life: left alone with the keys, an emergency radio and off you go

The scope and level of introduction for locums varied between municipalities. However, most locums had an introductory talk within a couple of hours of starting their contracts. The introduction lasted between one hour and half a day, and sometimes included activities such as how to access digital systems or a tour of the general practice. All locums had experienced poorly functioning digital systems, and brief and insufficient training. They therefore felt left to fend for themselves in an unfamiliar setting, as expressed by this participant:

“*I was a substitute for two weeks, and the municipality didn’t use any resources to welcome me… ‘Hello, welcome, here are the keys’. or just ‘The key’s under the mat’. You’re on the job as soon as you walk through the door. That’s my experience most of the time.*” *– Locum 6*

The municipality rarely provided any preparation in advance. In some rare cases, locums had brief meetings with the previous locum or received access to digital learning platforms to learn about local procedures. Since induction for locums was often not a priority, experienced locums described how they prepared themselves before a locum period. They read up on emergency medicine, contacted the municipality, and allocated enough time to familiarise themselves with the GP surgery, local geography and local emergency resources.

The locums wanted efficient induction procedures and considered these important for their well-being and ability to perform at work. They benefitted most from introductory programmes that included observations of office routines and practice, and training in administrative systems and the electronic patient record. This helped them to learn local systems, routines and workflow in the general practice in addition to making them feel more welcome.

### Theme 4: maintaining continuity in discontinuity

#### Barriers to continuity

Locums generally did not know their patients and had to assess treatment plans made by the previous GP. They had to rely on notes in the patient record, local staff and patients’ statements to obtain information about patients. Locums covering for absent GPs described this as ‘working on another person’s land’. This situation could complicate clinical decision-making, especially when locums found that they disagreed with the treatment prescribed by the previous doctors (GPs and locums). Several locums described their work as being akin to working in emergency primary care: unfamiliar patients (often from other GP lists), a focus on detecting obvious risks to patients, and reduced capacity to follow up patients afterwards:

“*A typical consultation is a bit like the emergency room. Where every new patient is a complete stranger. And then it’s just: ‘How can I help you?’ And then you start tidying up from there.*” *– Locum 4*

A lack of documentation in the patient record and delayed test results made it difficult to provide adequate care to patients. The locums had no information about patients with chronic diseases in need of further care and only became aware of them if patients booked an appointment themselves. Some locums were concerned about the cumulative effect of this disjointed patient care, since they had to rely on external factors such as the previous locum’s entries in the patient record or the patient’s own initiatives.

#### Strategies for maintaining continuity

A further problem was that locums were unsure about how their successor would follow up on their treatment. Several locums therefore stated that it was important to write detailed notes in the patient record, including treatment plans. Locums with repeated contracts booked their patients for a follow-up consultation for when they returned. Experienced locums reflected on their role in rural healthcare continuity, finding that they represented a break in continuity of care. To counteract this situation, they pointed out the importance of preparations. One experienced locum expressed the following:

*“I find it critical to have plenty of time for the consultation. You need time to read up on old patient journals, you need time to tune in to the patient, because you do not have 20 years of history with this patient.” – Locum 9*

Some locums negotiated long consultations into their contracts to enable them to spend more than 15 min with each patient. One locum explained how this extra time (30 min sessions) allowed them to review the patient record, which could reveal neglected chronic conditions in patients coming in for consultations for other medical issues and enable them to start treating these conditions:

*“Sometimes, I spend ten minutes reading up on the patients’ heart disease. Because I notice that there is a lot that needs sorting. Then the patient steps in with an ingrown toenail. We help him with the toe, at the same time encouraging them to book another appointment for the things i actually find important. To manage their diabetes and refer them to cardiologist as they have not had a check-up for years.” – Locum 8*

## Discussion

In this interview study, we have investigated the experiences of locum doctors working in rural general practice. These locums are usually recruited by third parties (agencies or former colleagues) until they develop a network of their own; early career locums mostly found it easier to use agencies. Locums were attracted to temporary work for the freedom it provided and the benefits involved. However, while early career locums often took the work to gain relevant work experience, experienced locums highlighted their desire for less stress and more patient-focused work than in their previous workplaces. All locums reported high expectations from municipalities to perform, often with little formal support. Early career locums sought advice and guidance to compensate for their limited experience. To maintain continuity of care, locums used longer consultations and wrote detailed notes in the patient record for the next locum. We will discuss our results in the light of the qualification requirements for locums and the implications for continuity of care.

### Locum work as way to mitigate issues in the regular healthcare employment

It has been consistently documented, on an international basis, that regular general practitioners report declining job satisfaction [30]. Norwegian GPs express dissatisfaction with increased workload and long workhours [2]. Some locums in our study had experiences with these negative developments while working as regular GPs; especially feelings of stress and guilt caused by having to balance their workload and responsibilities outside of work. Locum work seemed like a way to take control over work-life balance and mitigate some of the challenges associated with working in regular general practice.

In addition to increased workloads, end-of-career GPs report a detrimental change in general practice throughout their career, describing a trend with increased administrative work and less face-to-face clinical patient work and a reduction in the clinical scope of the GPs [31]. Rural and remote healthcare work might be a unique context for wide and challenging clinical practice, given the inherent need for specialist-generalist competency [32], and a slower tempo compared to urban general practice, enabling practitioners more time with face-to-face clinical work with patients, a sentiment voiced by several locums. Problems often associated with rural healthcare, such as professional isolation and long periods on-call [32], might also be mitigated by the temporary nature of locum work. However, locums as the main strategy for staffing is not sustainable and might reduce continuity of care for patients. Several of the locums we interviewed worked or had applied for regular GP positions in rural areas as a work-leave rotation doctor. Doctors using these schemes report to provide some of the same benefits as locums in terms of flexibility [26]. While still relatively high-cost services, it could, especially for rural and remote areas, be a way to provide more continuity of care and stabilize the turnover rate [26].

### The role of qualifications and the need for formal support

Ferguson et al. [33] and Stringer et al. [34] suggest that locums find themselves socially outside healthcare organisations, occupying an ambivalent position in relation to other healthcare workers, and thus becoming ‘others’. Consequently, locums, being seen as ‘others’, are not prioritised for accessing necessary formal support, such as introductory training and instruction [3,33]. This lack of prioritisation and inclusion reduced the ability of the locums in our study to adapt to the new work environment. This effectively reduces the safety of locum work and increases their need for support [3] Ferguson et al. [33] and Stringer et al. [34] recommend that ensuring social integration may be important to reduce the effect of ‘othering’.

The municipalities expect both experienced and inexperienced GPs to take on similar clinical workloads and perform equally complicated tasks; inexperienced GPs report struggling more with difficult consultations and confrontational patients [35]. Ignoring this difference in experience might increase the workload of local healthcare staff and the risk of patient harm [14]. In addition to this phenomenon, we are concerned about the lowered qualification requirements for short-term contracts. Only 28% of all locums have a specialisation in general practice, 58% have completed residency or the equivalent, while 14% have not completed residency [15]. The current regulations do not differentiate between levels of clinical experience. They enable municipalities to recruit locums without providing the support we would expect early career doctors to have during residency or specialisation in general practice [2]. This is in stark contrast to how other Scandinavian countries such as Sweden and Denmark set their qualification criteria for locum work.

Introductory programs which emphasise on-the-job skills training and co-worker observation could improve organisational social integration for early career professionals [36], and the locums in our study explicitly voiced an interest in such activities. Developing national guidelines and handbooks such as those NHS England has for locums [37] and organisations engaging with locums [38] could prove beneficial for the social integration of locums and mitigate some of the resource challenges associated with locum work. Additionally, guidance from regular staff for early career locums could reduce mental strain [39], increase patient safety [40] and reduce the need for informal support from local general practice staff. However, implementing new guidelines requires careful consideration. As in the UK, the staff of general practices were often unfamiliar with the established national guidelines for inducting locums [4,41].

### Locums and continuity

For locums, establishing interpersonal continuity with patients is rarely feasible within the timeframe of a locum contract, as such continuity is estimated to require one to five years, or four to five consultations with the same patient within a year [42]. The locums described several strategies to adjust to the uncertainty and lack of continuity by focusing on maintaining informational and longitudinal continuity through detailed entries in patient records and longer consultations. Hence, it is critical to ensure that locums have ready access to patient records and clearly understand how the digital infrastructure functions in a general practice. Access to digital learning platforms and time to learn these tools could be beneficial to prepare locums for local medical records.

Some locums used longer consultations to collect more detailed information about patients and their history. While patients prefer interpersonal continuity, this preference is reduced if there is effective informational continuity coupled with the perception by patients that the GP cares about them [43]. Longer consultations may be an indicator of increased patient-centred care [44,45] and quality of care [45]. Therefore, general practices could provide longer consultations, especially early in a locum contract, to increase the quality and continuity of care for patients.

Effective use of locums could support longitudinal care through access to care. However, continuity of care could further be improved by recruiting and retaining the same locums. Most of the locums in our study tended to find municipalities they favoured and returned to them repeatedly, even ending up in regular GP positions after some time as a locum. Hiring the same locums could reduce the workload associated with training and support and increase continuity of care for patients.

### Strengths and limitations

According to the model of information power [46], the sample size in a qualitative study should be considered in the light of the following factors: aim, specificity, theory, quality of the dialogue and analysis. In this study, we explored the experiences of GP locums working in rural Norway, a semi-narrow aim. Sample specificity was narrow, including experienced and early career locums with ongoing or recent contracts with rural municipalities. The inclusion of early career locums has been lacking in previous qualitative studies on locum work [47,48].

The in-depth interviews with open-ended questions provided strong dialogues that contained rich and broad data. The authors chose the two-to-one interview format to provide multiple perspectives during the initial eight interviews [49]. This approach to interviewing enabled a dynamic form of interview. It allowed the interviewers to benefit from each other’s strengths and competencies in the interviews and enabled immediate reflection and experience sharing afterwards. Furthermore, the team’s breadth of experience from rural primary health care practice and research facilitated reflection in the research team and added nuance and richness to the analysis. Reflexivity was developed by combining field notes, journaling and discussions within the research team. We did not apply any established theories to this study. Using relevant thematic theories could have sharpened our analytical focus.

There are some limitations to this study. Municipal administrations served as gatekeepers during recruitment. Therefore, we might not have captured the extremes of locum experiences within our sample, such as locums being accused of medical malpractice. Furthermore, the interviews were conducted digitally, with only some participants using cameras. There is also some loss of non-verbal communication in digital conference calls [50]. However, since we were interested in the locums’ experiences, this communication was of less relevance for the analysis at hand.

## Conclusion

To our knowledge, this is the first study to explore locum experiences in rural general practice, thus providing new perspectives on the phenomenon of temporary work in primary health care. Key features of locum work in rural areas are high expectations for performance, fragile continuity of care, a high degree of responsibility and limited formal support. There are no standard guidelines on how to introduce locums to Norwegian general practice, which is especially important for early career locums in need of support. In rural general practice, there is a need to improve the introduction and follow-up support of locums, to reduce the workload of general practice staff, and to improve the quality and continuity of care. It could be useful to look to similar healthcare system such, like Sweden’s, as they have formalised the use of non-specialised locums and incorporated support functions for early career locums. Furthermore, the results from our study suggest that rural locum work can be a way to mitigate the disadvantages associated with both urban and rural general practice and increasing the individual perceived benefits of general practice. Locums differ on what motivates the most for locum work. Healthcare managers and policy makers need to consider alternative ways of recruitment and retainment of both locums and regular personnel in the future.

## Implications

The knowledge generated from this study provides insights into temporary work and rural primary health care for clinicians, general practices, policy makers and researchers. Our results question the need for reduced qualification requirements for locum GPs. The qualification criteria as they stand today need to be modified. Furthermore, to support municipalities and general practices in hiring and introducing locums to GP work, national guidelines should be developed.

Through our analysis we highlight the role of municipal administrations as employers. Future qualitative studies should focus on exploring employers’ perspectives on efforts to recruit and retain locums. Further, studies should examine locum demographics and work patterns to better understand this group of employees within the job market. Studies should also compare the treatment patients receive from locums and regular GPs to determine any differences in the care provided.

## Supplementary Material

appendix_1_interviewguide.docx

## Collaborations

This study was performed in collaboration with the Norwegian Centre for Rural Medicine.
