# Supplementary material for: Motivations and challenges experienced among locums working in Norwegian rural general practice: a reflexive thematic analysis
Source: Scand J Prim Health Care. 2026 Jun 1;44(1):2679616. doi: 10.1080/02813432.2026.2679616 (PMC13231821; doi:10.1080/02813432.2026.2679616)
Supplement: appendix_1_interviewguide.docx [file IPRI_A_2679616_SM8548.docx]

**Appendix 1: Interview guide developed and used for the semi-structured interviews**

| **Primary questions** | **Follow-up questions** |
| --- | --- |
| What are your motivations for working as a locum? | What made you choose this specific municipality? |
|  | What motivated you to choose rural general practice? |
|  | What are your plans in terms of work/career? |
| How were you recruited to the position? | Could you tell me about the recruitment process? |
| How were you received at the general practitioner’s surgery? | How were you received by the municipality? |
|  | How were you received by the local community? |
|  | Who oversaw your introductory training? |
| Could you describe a typical day as a locum general practitioner? | Is there anything that surprised you when working as a locum? |
|  | Have you received comments or feedback from patients, family members or colleagues? |
|  | Could you tell me about how you work regarding sick notes, referrals and prescriptions? |
|  | Could you tell me about some of the locums you have been working with? |
|  | Do you have any thoughts on how locums best can contribute to patient safety and quality of care? |
|  | If you had the opportunity, what kinds of changes would you introduce to increase patient safety and quality of care in the context of locum work? |
| Could you tell me about the terms and conditions of your contract? | How was your living situation throughout the contract period? |
|  | Could you tell me about how you were compensated? |
|  | Is your contract with the municipality, the practice, an agency or another body? |
